# The co-use of conventional drugs and herbs among patients in Norwegian general practice: a cross-sectional study

**DOI:** 10.1186/1472-6882-13-295

**Published:** 2013-10-30

**Authors:** Ane Djuv, Odd Georg Nilsen, Aslak Steinsbekk

**Affiliations:** 1 Department of Cancer Research and Molecular Medicine, Faculty of Medicine, Norwegian University of Science and Technology, Trondheim, Norway; 2 St. Olavs Hospital, Trondheim University Hospital, Trondheim, Norway; 3 Department of Public Health and General Practice, Norwegian University of Science and Technology, Trondheim, Norway

**Keywords:** Herb-drug interaction, General practice, Safety, herbal use, Disclosure, Complementary therapy, Elderly, Polyherbacy, Polypharmacy

## Abstract

**Background:**

Different patient groups are known to use herbal remedies and conventional drugs concomitantly (co-use). This poses a potential risk of herb-drug interaction through altering the drug’s pharmacokinetics or pharmacodynamics. Little is known about co-use among patients in general practice. The primary aim of this study was to compare patients in general practice that co-use herbal remedies and conventional drugs with those who do not. The secondary aim was to register the herb-drug combinations with potential clinical relevant interactions among the co-users.

**Method:**

A questionnaire based cross-sectional study conducted in the autumn 2011 in a general practice office with four general practitioners (GPs) and one intern in Western Norway. Adults >18 years who came for an office visit were invited. The questionnaire asked about demographics, herbal use, conventional drug use and communication about herbal use. Multivariable logistic regression was used to compare co-users to the other patients.

**Results:**

Of the 381 patients who completed the questionnaire, the prevalence of herbal use was 44%, with bilberry (41%), green tea (31%), garlic (27%), Aloe vera (26%) and echinacea (18%) as the most frequently used. Among those using conventional drugs regularly, 108 (45%) co-used herbs. Close to 40% of patients on anticoagulants co-used herbs, with garlic and bilberry as the most frequent herbs. Compared to all other patients, co-users had significantly (p < 0.05) increased odds to be female (adjOR 2.0), age above 70 years (adjOR 3.3), use herbs to treat an illness (adjOR 4.2), use two or more herbs (polyherbacy, adjOR 12.1) and having experienced adverse effects of herbal use (adjOR 37.5). Co-use was also associated with use of analgesics or dermatological drugs (adjOR 5.1 and 7.9 respectively). Three out of four patients did not discuss herbal use with any health care professional.

**Conclusion:**

A sizable proportion of the GP patients co-used herbs with conventional drugs, also combinations with reported interaction potential or additive effects like anticoagulants and garlic. The low disclosure of herbal use to their GP, polyherbacy and the risk of interactions in vulnerable groups like elderly and chronically ill patients, warrant increased awareness among GPs.

## Background

In the last two decades there has been a considerable increase in the herbal remedy market [1,2]. Interactions between herbal remedies and drugs have been put on the agenda and received increased attention [3,4]. Both serious and less serious adverse interactions have been reported e.g. between the drug cyclosporine and St. Johns wort (*Hypericum perforatum*), and between drugs like warfarin or aspirin which are reported to interact with a range of herbs like garlic (*Allium sativum*), cranberry (*Vaccinium oxycoccos*), *Ginkgo Biloba*, ginger (*Zingiber officinale*) and grape fruit (*Citrus paradisi*) [5-9]. Co-use of herbs and drugs might alter the drug’s pharmacokinetics and/or pharmacodynamics, hence causing unexpected adverse effects of the drug [10-13].

Studies have reported extensive use (40-56%) of herbs in the general population [14-16]. The 2007 National Health Interview Survey, USA, reported that nearly 20% of the general population were using herbs [17]. The typical herb user was female, aged 30 to 69 years, with higher education or hospitalized in the last year [17]. Forty-one per cent of USA adults reported the use of herbal remedies to self-treat before seeking medical care from a physician [14].

Only 50% of herb users inform their physician about it [14]. In addition, the health care professionals rarely ask the patients about the use of herbs or other types of complementary and alternative medicine [18]. ”The doctor did not ask” is the common phrase explaining the lack of communication [19]. The general practitioners (GPs) also tend to underestimate the use [18]. It is therefore important to have knowledge about the characteristics of herb users in general and co-users in particular to make health professionals more aware.

It is reported that up to 40% in various patient groups co-administrate herbal remedies and drugs [20-22]. One study found that 40% of pregnant women used herbal remedies and about 85% of these co-used conventional drugs [21]. The use of herbal remedies among adults with cancer is reported to be between 30-55% [15,23] and one study found that almost 40% co-use herbal remedies and chemotherapy [24]. Elderly patients have more poly-pharmacy problems and are more vulnerable to interactions because of altered pharmacokinetics and decreased health in general [25]. Considering that 13-47% of elderly patients report to consume herbs [26,27] and 31- 75% of these co-use herbs and prescribed conventional drugs [28,29], the risk of adverse interactions might be high. About 50% of the general population have one or more chronic conditions and as the elderly, they have a high care rate and poly-pharmacy (50%) [30]. They also tend to use more herbal remedies, which increase the possibility of herb-drug interactions [31].

Despite the large reported use of herbs and co-use of herbs and conventional drugs in the general population and in various patient groups, few studies have been performed among patients in primary care and general practice in particular. About 40% of the patients in primary care clinics in USA believed that taking prescription medications and herbal remedies together was more effective than taking either alone and nearly 50% of the herb users co-used drugs [32]. An Israeli study on co-use among patients in general practitioner’s offices, reported 36% of herbal use and approximately 30% were co-users [33]. GPs are the first medical contact within the health care system, dealing with all health problems both acute and chronic [34]. Given the nature of general practice, the few studies are somewhat surprising.

The primary aim of this study was to compare patients in a general practice in Norway that co-use herbal remedies and drugs with those who do not, with regards to demographics, types of drugs and herbs used, reason for use and communication with health care professionals about this use. The second aim was to register the herb-drug combinations with potential clinical relevant interactions among the co-users.

## Methods

This was a questionnaire based cross-sectional study. The survey took place in a general practitioners office with four GPs and one intern physician situated in the city centre of a middle sized town with nearly 70 000 inhabitants on the west coast of Norway. About 6000 patients were on the GPs list at the time of the data collection. The data collection took place during 5 weeks in the autumn 2011 (11th November till 15th of December). The study was approved by the Regional Committee for Research Ethics in South-eastern Norway.

### Participants and recruitment

The inclusion criteria were patients 18 years old or older, having an office consultation with a GP and who were able to read and understand the questionnaire.

The questionnaire was first made available to the patients in the waiting area for self-inclusion, but after a short time the recruitment was done by the staff in the reception. The reception staff was instructed to consecutively ask the patients who contacted them when they prior or after the GP consultation whether they would be interested in taking part in the survey and gave the questionnaire to those who said yes. It was not systematically registered how many said no, but according to the staff this was about half of the patients. The first page of the questionnaire informed the patient about the project, its objectives and the handling of their information. In addition, information was given on wall posters in the waiting area. The reception staff assisted the participants with the questionnaire whenever needed. The patients were asked to return the questionnaire to the reception or by mail in pre-addressed and pre-paid envelopes. Their answers were anonymous. A completed questionnaire was interpreted as informed consent.

### Questionnaire

The questionnaire included questions about herbal use, drug use and communication about herbal use and was based on a questionnaire previously used among cancer patients in an outpatient clinic in Central Norway [24].

The questionnaire was divided into three parts. The first part contained questions about demographic data (Table 1) and about conventional drugs used regularly from a predefined list of 25 drug-categories with possibilities to add other drugs (Table 2). The drug categories covered most of the regularly prescribed drugs based on data from the Norwegian Institute of Public Health and were exemplified with common Norwegian sales name to make them recognizable for the patients [35].

**Table 1:** Demographics of all respondents according to herb and drug use; comparison of conventional and non-conventional drug users among herbal users and non-herbal-users, and comparison of co-users with non-co-users (N = 381)

				**Current herbal user?**						**Co-user**
				**Yes**			**No**			
				**Regular drug user?**			**Regular drug user?**			
		***n (%)***		**Yes**	**No**	***p-value***^***A***^	**Yes**	**No**	***p-value***^***A***^	***p-value***^***C***^
				**(Co-user)**	**(Herb-only user)**		**(Drug-only user)**	**(Non-user)**		
Gender	Male	124	(33)	18%	11%	*0.551*	39%	32%	*0.085*	0.001^*B*^
	Female	249	(65)	34%	16%		33%	16%		
Age grouped	<30	50	(13)	20%	20%	*<0.001*^*B*^	18%	42%	*<0.001*^*B*^	*0.008*^*B*^
	30-39	58	(15)	24%	21%		24%	31%		
	40-49	52	(14)	12%	29%		37%	23%		
	50-59	65	(17)	34%	15%		34%	17%		
	60-69	71	(19)	38%	4%		35%	23%		
	>70	76	(20)	37%	7%		54%	3%		
Education	Compulsory	71	(19)	32%	8%	*0.178*	46%	13%	*0.010*^*B*^	*0.611*
	Middle level	170	(45)	28%	15%		29%	27%		
	University	129	(34)	26%	18%		36%	20%		
Employment	Employed/Off sick	233	(61)	21%	20%	*<0.001*^*B*^	30%	29%	*<0.001*^*B*^	*<0.001*^*B*^
	Disability or retirement pension	129	(34)	40%	5%		47%	9%		
	Unemployed/Home	12	(3)	58%	17%		8%	17%		
Herbal use	Never	152	(40)				63%	37%	*0.533*	
	Earlier	60	(16)				58%	42%		
	Present	164	(44)	66%	34%	*0.450*				
Recommended to use herbs by (n = 110):^D^	Friends or family	75	(68)	61%	39%	*1.000*				
	The Physician	5	(5)	80%	20%	*0.647*				
	The shop or pharmacy	32	(24)	59%	41%	*0.830*				
	Read about it in magazines or internet	35	(6)	69%	31%	*0.401*				
	The alternative therapist	7	(32)	43%	57%	*0.424*				
	Other	3	(6)	33%	67%	*0.152*				
Reasons for herb use (n = 111):^D^	Better life expectancies	47	(3)	62%	38%	*1.000*				
	Strengthen the immune system	79	(42)	58%	42%	*0.391*				
	Defeat an illness	18	(71)	89%	11%	*0.008*^*B*^				
	Better than nothing	7	(16)	29%	71%	*0.106*				
	Pain relief	4	(6)	50%	50%	*1.000*				
	Other	3	(4)	100%	0%	*0.280*				
Reasons for not using herbs (n = 177):	Never considered it	69	(3)				51%	49%	0.002^*B*^	
	No need/Satisfied with the treatment I get	52	(39)				58%	42%		
	Do not believe in it/ seems unsafe	56	(29)				80%	20%		

^A^P-value for comparison of conventional drug user with not conventional drug user. Analysed with Pearson Chi-Square or Fisher exact test.

^B^p < 0.05.

^C^P-value for comparison of co-users with non-co-users. Analysed with Pearson Chi-Square or Fisher exact test.

^D^Multiple answers were possible.

**Table 2:** **Total number and proportion of herb users for the different drug categories (*N*** **= 239)**

**Drugs (ATC group)**	**Total *n *** **(%)**	**Proportion of co-users**	**p-value**^**A**^
Against gastrointestinal conditions (A01-09)	15 (6)	60%	*0.288*
Analgesics (M01A, N02B)	55 (23)	58%	*0.031*^*B*^
Anti-infectives (G01, J01-05)	2 (1)	50%	*1.000*
Anticoagulants (B01)	88 (37)	36%	*0.043*^*B*^
Antidepressants (N06)	20 (8)	50%	*0.815*
Antidiabetics (A10)	23 (10)	52%	*0.515*
Antihistamines (R06)	25 (10)	32%	*0.204*
Antihyperlipidemic agents (C10)	81 (34)	47%	*0.784*
Antihypertensives and diuretics (C02-C03, C07-09)	107 (45)	45%	*1.000*
Anti-menopausal and anticonceptives (G01-03)	21 (9)	48%	*0.823*
Antirheumatic (L01, L04, M01-04)	28 (12)	39%	*0.550*
Antiseizure, triptanes and central stimulating drugs (N02C, N03, N06B)	20 (8)	45%	*1.000*
Chemotherapeutic drugs (L01-04)	5 (2)	60%	*0.660*
Dermal drugs (D01-11, C05)	14 (6)	64%	*0.171*
Drugs against urogenital and prostate disorders (G04)	11 (5)	55%	*0.551*
Ocular drugs (S01)	9 (4)	44%	*1.000*
Respiratory drugs (R01-05, 07, H02)	20 (8)	45%	*1.000*
Sedatives and Antipsychotics (N05)	39 (16)	49%	*0.726*
Strong analgesics (N02A, N07BC)	3 (1)	33%	*1.000*
Thyroids and antithyroids (H03)	26 (11)	54%	*0.406*
Vasodilators and cardiac glycosides (C01)	5 (2)	60%	*0.660*
Other drugs total	14 (6)	57%	*0.413*

^A^P-value for comparison of herbal user or non-user for each drug category, analysed with Pearson Chi-Square or Fisher’s exact test if the number of total users was below five.

^B^Significantly different with p < 0.05.

The second part asked about herbal use from a predefined list of the 24 most common herbs sold in Norway and the frequency of use [36] (Table 3). In addition, supplements (e.g. multivitamins) and an extra space for other herbs were also included. Only those products defined as herbs (herbal substances, herbal preparations or herbal medicinal products) were included in the analysis [37]. Herb users were defined as those answering that they used herbs daily, weekly, monthly, less than monthly or periodically. Non-users were defined as those answering that they do not use herbs now, but have used herbs earlier or never used.

**Table 3:** **Types of herbs used and proportion of co-users of conventional drugs and herbs (*N*** **= 164)**

**Herbs**^**E**^	**Total use *n *** **(%)**	**Proportion of co-users**	***p-value***^***A***^
Aloe vera^F^	42 (26)	55%	*0.091*
Apple vinegar	10 (6)	70%	*1.000*
Bilberry^G^	68 (41)	68%	*0.740*
Cranberry	26 (16)	77%	*0.261*
Echinacea	29 (18)	55%	*0.200*
Essiac	1 (1)	0%	*0.341*
Garlic	44 (27)	61%	*0.578*
Ginger	18 (11)	50%	*0.186*
Ginkgo Biloba	4 (2)	100%	*0.300*
Ginseng	15 (9)	67%	*1.000*
GLA/Evening Primrose oil	8 (5)	50%	*0.447*
Golden root	10 (6)	80%	*0.497*
Grapefruit	13 (8)	46%	*0.135*
Green tea	51 (31)	65%	*0.860*
Misteltoe	1 (1)	0%	*0.341*
Nattokinase	2 (1)	100%	*0.548*
Noni juice	5 (3)	20%	*0.047*^*B*^
Soya extract	4 (2)	75%	*1.000*
Valeriana	4 (2)	75%	*1.000*
Others in total^C^	15 (9)	67%	*0.480*
- Other: Anthocyanin^D^	4 (2)	75%	*1.000*
- Other: Saw Palmetto	2 (1)	100%	*0.548*

^A^P-value for comparison of co- users with herb users alone. Analysed with Pearson Chi-Square or Fisher exact test.

^B^Significantly different with p < 0.05.

^C^Herbs added by the respondent to the open question about other herbs they used.

^D^Anthocyanin extracted from outer layers of bilberry and blackcurrant.

^E^Herbs included in the questionnaire with no users: Shark cartilage and St. Johns wort.

^F^May include either topical or oral Aloe vera use.

^G^May include both bilberry (V. myrtillus) and/or blueberry (V.cyanococcus) due to confused with one another.

In the last part of the questionnaire the communication between the patient and health care professionals, motives for use or no use and who recommended use of herbs were obtained (Tables 1 and 4). In addition, they were asked about any side effects of their herbal use and approximately monthly costs.

**Table 4:** Communication with health care professionals, adverse effects and monthly costs of herbs among current herb users and proportion of co-users of conventional drugs and herbs

		**Total *n *** **(%)**	**Proportion of co-users**	***p-value***^***A***^
Communication about herb use with (n = 146):^C^	Physician^D^	27 (18)	74%	*0.269*
	Other	10 (7)	80%	*0.324*
	Never discussed	113 (77)	59%	*0.104*
The health care providers response to herb use (n = 167):	Not discussed	134 (80)	63%	*0.463*
	Encouraged use	12 (7)	83%	
	Discouraged use^E^	7 (4)	57%	
	Neutral/indifferent	14 (8)	71%	
Reasons for never discussing herb use with health care professionals (n = 110):	I was never asked	50 (45)	56%	*0.723*
	Afraid of the response^F^	23 (21)	65%	
	Only my own concern/confidential	34 (31)	62%	
	Uncertain of the herbal effect	3 (3)	33%	
Experienced adverse effects of herbs (n = 120)?	Yes	8 (7)	100%	*0.026*^*B*^
	No	112 (93)	61%	
Costs of herb use per month (Euro^G^)	Mean (SD, range)	36.6 (29.0, 0.4-205)	40.4 (34.8, 0.4-205)	*0.337*
Cost range, NOK (Euro) (n = 88)	1-199 (0.1-27.2)	24 (27)	71%	*0.330*
	200-399 (27.3-54.5)	47 (53)	57%	
	400-599 (54.6-81.8)	9 (10)	56%	
	>600 (>82.0)	8 (9)	88%	

^A^P-value for comparison of communication, motives for herbal use, adverse effects and costs between co-users of drugs and herbs and not co-users. Analysed with Pearson Chi-Square or Fisher’s exact test given the number of total users were below five.

^B^Significantly different with p < 0.05.

^C^Multiple answers were possible.

^D^Includes GPs, regular GPs (family doctors) and hospital physicians.

^E^A merge of the responses «warned about the risk” and “discouraged use”.

^F^A merge of the responses “I was afraid of not getting acknowledgement for my choice”, “I was afraid they got dissatisfied” and “I was afraid of being rejected”.

^G^Converted from NOK to Euro. Exchange rate retrieved 23.11.2012 at 09.12 AM (1 Euro = 7.32 NOK).

### Statistics

To find the total number of consultations in the GP practice during the 5 weeks data collection period, and the age and gender distribution of these patients, a report module of the electronic health record system was used (WinMed 2.12r Statistics, CompuGroup Medical Norway AS, Lysaker, Norway).

Pearson’s Chi-square was used for bivariable analyses of categorical data, like the differences between users and non-users of conventional drugs. In analyses that included less than 5 cases in a cell, the Fisher exact test was used. For multivariable analysis to disclose any associations between co-users and other variables, binary logistic regression analysis (adjusted odds ratio, adjOR) was used. All variables with p-values <0.2 in bivariable analysis were included in the regression analysis. In addition, a separate multivariable logistic regression model were used to compare co-users with drug-only users including variables with a p-value <0.2 in the bivariable analyses of variables that both co-users and drug-only users answered (from Table 1, 2 and 3). P-values < 0.05 were considered as statistically significant. Tendencies were ascribed for p-values between 0.05 and 0.10. The statistics analysis was done using SPSS 19.0 (SPSS, Chicago, IL, USA).

## Results

The total number of patients having consultation in the GP office during the five weeks of data collection was 1652. Fifty-seven per cent of these were females. The average age was 54.5 years, with 25% being 70 years old or above. The other age groups, grouped as shown in Table 1, were evenly distributed in the range 13-17% of the total number patients.

Four-hundred and two questionnaires were distributed and 381 were returned. Of the 381 respondents, 67% were females, the average age was 52.5 years (SD = 18.11, range 18–92) and 20% were 70 years old or above (Table 1). About 35% had higher education and 61% were employed or on sick leave (off sick). Nearly two out of three (63%) used conventional drugs regularly.

Nearly half of the patients used multivitamins or supplements (data not shown). A total of 164 (44%) patients were currently using herbs, and there was a significantly higher proportion of women using herbs compared to men (51% of all female patients vs. 29% of all male patients, p < 0.001, Table 1). Elderly above 70 years old, had a significant higher herbal use with 91% using herbs alone (herb-only user) or co-using with drugs compared to the youngest patients (p < 0.001). A total of 74 (20%) of the patients were using two or more herbs (polyherbacy) and about 80% of those were women (p < 0.001, data not shown). For the other demographic variables there was no significant differences with regard to herb use (data not shown).

Among those using conventional drugs, 108 (45%) also used herbs (co-users). Significant differences were seen between the genders, age and occupational groups in regard to co-use of drugs and herbs (Table 1). Compared to men, females co-used significantly more drugs and herbs (18% vs. 34%, p = 0.001). More than one of every three patients older than 50 years were co-users and this was significantly more than for younger patients (p = 0.008). Those employed co-used significantly less than those not employed (p < 0.001).

Friends or family were those most frequently recommending herbal use (68%), followed by magazines or internet (32%), the shop or pharmacy (29%, Table 1). Significantly more of the co-users than non-co-users, used herbs with the intention to treat an illness (89% vs 11%, p = 0.008). The most common reasons for no use were “Never considered it” (39%) and “Do not believe in it/ Seems unsafe” (32%).

Among those who used herbs, bilberry (*Vacciunum myrtillus*, 41%), green tea (*Camelia sinensis,* 31%), garlic (*Allium sativum*, 27%), Aloe vera (*Aloe barbadensis,* 26%) and echinacea/purple coneflower (*Echinacea purpurea*, 18%) were the most commonly used herbs (Table 3). For nearly all the types of herbs used, there were no significant difference between the types of herbs used when comparing those who co-used conventional drugs and those who did not use conventional drug. Among the five most commonly used herbs (18% or more of the users), those who co-used conventional drugs tended to use more Aloe vera than not conventional drug users (p = 0.091). In addition, almost two of three (63%) of the polyherbacy patients were also using conventional drugs (p < 0.001).

For nearly all types of conventional drugs used there were no significant differences between herb users and non-users (Table 2). The only significant difference was higher use of herbs among those using analgesics (60% used herbs vs 40% did not, p = 0.031) or anticoagulants (36% used herbs vs 64% did not, p = 0.043).

A total of 255 different herb-drug combinations were registered (Additional file 1: Table S1). Of these, 18 were identified of being at risk of clinical relevant interactions (these are highlighted with numbers in bold, Additional file 1: Table S1). Antihypertensives and diuretics were the largest drug categories in regard to number of combinations with different herbs (n = 20) followed by analgesics (n = 19), antihyperlipidemic agents (n = 19) and thyroid- or antithyroid hormones (n = 17) (Additional file 1: Table S1). Bilberry (n = 21), green tea (n = 20) and cranberry (n = 20) were the herbs with the highest number of combinations with drugs. The most common combinations were seen between bilberry and antihypertensives (n = 24), anticoagulants (n = 18) or analgesics (n = 15) (Additional file 1: Table S1). Green tea and garlic had also high number of co-use for these drugs.

Nearly 80% of the herb users did not discuss herbal use with any health care professional. The majority (80%) of those were co-users (p = 0.104) (Table 4.). The most common health care professional the patients discussed their herbal use with was the GP (15%, data not shown). Of those discussing herbal use with their GP, about 80% were co-using conventional drugs (p = 0.156). The response from the GP on disclosure of co-use differed from encouraging continued use (32%), neutral response (32%) and discouraged (14%) herbal use (p = 0.815, data not shown).

Only non-co-users had been warned about risks with herb use (data not shown), while 83% of those being encouraged to continued use were co-users (p = 0.463). The most common reason for no communication was “I was never asked” (45%, p = 0.723).

All of those who had experienced adverse effects of herbs were co-users (7%, n = 8, p = 0.020). The herbs most frequently used by those experiencing adverse effects were garlic (n = 5), bilberry (n = 4), green tea (n = 4) and ginger (n = 3). The most common drugs co-used with herbs of this group were anticoagulants (33%) sedatives (33%) and antihypertensives (22%). Abdominal pain, diarrhea and emesis (33%) or dizziness (22%) was the most common reported effects.

### Multivariable analysis

A total of 17 variables were included in the binary logistic regression analysis comparing co-users to all other patients. Of these, seven variables were significantly (p < 0.05) associated with co-use of herbs and conventional drugs (Table 5), with an increased odds for co-users to be female (adjOR 2.0), above 70 years (adjOR 3.3), wanting to treat an illness (adjOR 4.2), using several herbs (polyherbacy, adjOR 12.1) and experience adverse effects (adjOR 37.5). Increased levels of co-use were also associated with use of analgesics or dermatological drugs (adjOR 5.1 and 7.9 respectively). Being between 40 and 49 years old decreased the odds of being a co-user (adjOR 0.2).

**Table 5:** Adjusted odds ratio (adjOR) with 95% confidence intervals (95%C.l.) from multivariate regression models for co-use of herbal remedies and conventional drugs.

			**95% C.I.**		
		**adjOR**	**Lower**	**Upper**	***p-value***^**A**^
Gender	Female vs male	2.0	1.0	4.0	*0.043*^B^
Age grouped	Age < 30 vs:				*0.000*^B^
	-30-39	0.8	0.3	2.6	*0.715*
	-40-49	0.2	0.0	0.9	*0.034*^B^
	-50-59	1.3	0.4	3.8	*0.665*
	-60-69	2.8	1.0	8.3	*0.058*
	->70	3.3	1.2	9.3	*0.023*^B^
Reasons for herb use	Treat an illness	4.2	1.3	13.4	*0.015*^B^
Drugs	Analgesics	5.1	2.4	10.7	*0.000*^B^
	Dermatological drugs	7.9	2.0	30.8	*0.003*^B^
Adverse effects of the herbal remedy	Yes vs No:	37.5	2.8	503.4	*0.006*^B^
Polyherbacy	None or one herb vs >2 herbs:	12.1	5.8	25.4	*0.000*^B^

^A^P-value for multivariable logistic regression with co-use as the dependent variable. Analysed with regression analysis and Forward method in SPSS.

^B^Significantly different with p < 0.05.

In the sub-analysis of co-user vs. drug-only users, the model included gender, and use of anticoagulants, analgesics and dermal drugs (data not shown). Those who co-used drugs and herbs tended (p < 0.100) to be female (adjOR 1.9) and use analgesics (adjOR 1.7) compared to drug-only users.

## Discussion

A total of 29% of GP patients in this study co-used herbs and conventional drugs. The co-use was associated with female gender, increasing age above 50 years, using herbs to treat an illness, polyherbacy, use of analgesics or dermatological drugs and having experienced adverse effects from herbs.

### Strengths and limitations

One of the limitations of this study was that it is a cross sectional study, meaning that no causal relationship can be identified. In addition, the study took place in one GP clinic in a middle sized town on the west coast of Norway. It thus might not be representative for other populations, but the patients visiting the practice are similar to other GP patients in Norway [38]. Although those taking part in this study were representative for all patients visiting the GP practice during the period of the survey, those using herbs might also be more positive to contribute to such a study than non-herb users. This would give an overestimation in the prevalence of herb users. However, this would also be the same for other studies investigating herbal use, and would not hamper the comparison with these. All data are self-reported and inaccuracies in the reported use of herbs and drugs must be taken into consideration. Still, the latter was minimized by handing out lists of the most common drugs in familiar groups with examples of the most common sales name of the different drugs.

### Herbal use

The prevalence of herbal use of 44% is somewhat higher compared to other findings from general practice/family doctors (22-36%) [32,33]. Our prevalence is surprisingly close to the findings from a Norwegian cancer patient clinic where a similar questionnaire was used (46%) [24]. It is also in range of the prevalence of studies of the general population from other countries. The 2007 National Health Interview Survey, USA, reported of nearly 20% herbal use in the general population [17]. However, both the Czech and Saudi-Arabian population reports of higher herbal use (50-57%) compared to the USA population [29,39]. Thus, the prevalence might vary between countries and ethnic groups [27].

Few patients were recommended herbal use by the pharmacy or a physician. As reported by other papers, friends or family are the common sources for herbal recommendation or information [14,23].

Bilberry, green tea, Aloe vera, garlic and echinacea were the most commonly used herbs among the patients. Except from bilberry, all other herbs are also frequently reported by others [11,17,40,41]. A sub-analysis of the reason for using bilberry revealed that it was used largely to strengthen the immune system (84% of bilberry user gave this as the reason). The use of bilberry might have been influenced by heavy marketing as a “super-food” [42].

Overall, every third patient in this study co-used drugs and herbal remedies. Reported co-use from GP’s offices in Israel in 2004 was lower (12%) [33], however, up-to-date numbers from GP practice are lacking. The co-use is in line with the co-use reported for patient groups like pregnant women (34%) and somewhat lower than reported for the cancer patients (30-55%) [15,21,23]. Thus, our findings are in line with earlier reported co-use for patient groups, and the prevalence of co-use seems to be similar across different populations.

### Characteristics of co-users

Based on the high co-use of drugs and herbs, drug users are at high risk of clinically relevant interactions [3,43]. As expected, increasing age above 50 years was associated with a higher co-use compared to the younger patients in our study (nearly 40% of those >70 years old were co-users). Earlier studies report of co-use among elderly from 32-42% [25,44,45]. Cohen et al. found co-use of 24% among geriatric patients, and 52% of them co-using with anticoagulants [46]. Elderly patients are an exposed group because of increasing poly-pharmacy, reduced general health and altered drug metabolism [25,47]. They have a lower tolerance for alterations in the pharmacokinetics or pharmacodynamics, which might have serious consequences [3,4,8]. In addition, females, or those taking two or more herbs, were both significantly associated with co-use in this study. Females are reported in several other papers as the most common user of herbal remedies, thus not in particular as co-users [48].

The most frequent co-use of drugs in this study was with bilberry, green tea, garlic, Aloe vera and cranberry. Bilberry is abounded of antioxidants and has been reported to have anti-inflammatory activity [49]. A recent case report indicates an interaction between bilberry and warfarin that induces rectal bleeding [50], however, few interaction data are published on this herb. Thus, attention should be paid to the intake of bilberry in patients taking antiplatelet or anticoagulant drugs. Garlic might have antiplatelet activity and should thus, be used with care together with antiplatelet drugs like warfarin [11,51]. Excessive bleeding has been reported in patients co-using warfarin and garlic, a patient group frequently using garlic [52]. Aloe vera might cause potassium depletion or affect cardiac glycosides and is advised not to be used together with heart medication [11]. However, no *in vitro* or *in vivo* pharmacological interactions have yet been established [11,53,54]. Cranberry is reported to interact with warfarin, increasing International Normalized ratio (INR) values by 30% [9], but an randomized controlled trial concluded with minor risks for significant interactions in humans [55]. Some reports state, however, that garlic, green tea, Aloe vera and cranberry in general seem to have a low drug interaction risk [12,55].

Those on regular analgesics or dermal drugs were significantly associated with co-use. NSAIDs (e.g. Aspirin) is known to interact with many herbs (e.g. ginkgo, garlic, ginger, bilberry, ginseng) and a recent study shows decreased *in vitro* metabolism of paracetamol when co-used with *Coriolus versicolor* used in traditional Chinese herbal medicine [8,56,57]. Keeping in mind that nearly half of the co-users used two or more herbs, the risk of interactions or additive effects are present.

In the present study, herbal adverse effects were only reported by co-users (7%). In a recent paper from Beirut as much as 60% of the co-users reported some sort of adverse effects [58,59]. Although our reported prevalence is low, those reporting adverse effects were using herbs with reported additive effects (e.g. anticoagulants and garlic) [11]. Still, the numbers are too low to draw any firm conclusions.

### Herb-drug interactions at risk

There were identified 255 different drug-group and herb combinations (Additional file 1: Table S1). Of these, 18 were identified of being at risk of clinically relevant interactions (in bold, Additional file 1: Table S1) on the basis of clinical trials, case reports or theoretical interactions extrapolated from clinical data [12,60]. Anticoagulants (e.g. warfarin) were co-used with garlic (*Allium sativum*), cranberry (*Vaccinium oxycoccos*), ginger (*Zingiber officinale*), ginseng (*Panax ginseng*), grape fruit juice (*Citrus paradisi*) and saw palmetto (*Serenoa repens*), all interacting with anticoagulants increasing the risk of adverse effects (e.g. increased haemorrhage) [6,8,9,13,20,52].

Antihypertensives and diuretics were the largest drug categories in regard to number of combinations with different herbal remedies in the present study, having interaction potential with ginseng or grapefruit juice [8]. Ginseng is also reported to interact with antidiabetics, cardiac glycosides, antidiarrheal agents and antidepressants [8,60]. Co-use of garlic with NSAIDs, anti-retroviral therapy or antidepressants have also been reported to give clinically relevant interactions [8,60]. In general co-use of these herbs with anticoagulants or other cardiovascular drugs should be discouraged or closely monitored for adverse effects/INR [11,52]. Co-use should especially be closely monitored or even discouraged among the elderly [61]. Anti-constipation drugs or antidiabetic agents should not be consumed with Aloe vera (*Aloe barbadensis*) because of additive effects and the same has been shown for valeriana (*Valeriana officinalis*) co-used with antidepressants [60,62].

The duration or amount of herb use and the way of administration of the herb (i.e. oral, topical) was not covered in this study and would have given us more information whether the herb-drug interaction was clinically relevant. Aloe vera used as juice ingested orally in large daily doses has a much higher interaction potential contra Aloe vera used topically against skin burns, although dermal absorption cannot be excluded. Some of the herbs are ingested as foods like garlic and grapefruit and will in general not be a problem, unless used in excessive amounts.

### GPs needs to ask all patients

The majority of herb users did not discuss their use of herbs with any health care professional and only 15% discussed herbal use with their GP. For those on conventional drugs, having a chronic illness and thus having a closer relationship to their GP, one should expect a higher willingness to share information about their herbal use. As the most common reason for not communicating about the subject is “I was never asked”, there are strong indications that patients are waiting for the GPs to be the one to take initiative in these matters. Although there are some characteristics of the co-users (female, elderly, use of certain drug groups etc.), there are unfortunately no specific variable that in our opinion can be used by the GP to pin-point co-users. The GP should therefore routinely ask all their patients about use of herbal remedies in order to identify potential harmful co-use.

## Conclusion

The high percentage of herbal co-use among patients using conventional drugs in general practice, and the relation between increasing co-use with increasing age and comorbidity, makes general practice an arena where co-use should be discovered. Given the under-communication with GPs about co-use, it is difficult to prevent unwanted adverse effects and interactions. In order to monitor co-use, all GPs should ask their patients routinely to disclose their use of herbs.

## Abbreviations

GP: General practitioner; INR: International normalized ratio; NSAID: Nonsteroidal anti-inflammatory drugs; RCT: Randomized controlled trial; USA: United States of America.

## Competing interests

The authors declare that they have no competing interests.

## Authors’ contributions

AD has made substantial contributions to conception and design of the study, did the data collection, performed the statistical analysis and interpretation of data, and the drafting and revising of the manuscript. OGN has been given final approval of the questionnaire and been involved in revising the manuscript critically for important intellectual content. AS has made contributions to conception and design, the statistical analysis and interpretation of data and was involved in revising the manuscript critically for important intellectual content. All authors read and approved the final manuscript.

## Authors’ information

AD is physician and is working as PhD student at Department of Cancer Research and Molecular Medicine and St. Olavs Hospital, Trondheim University Hospital. OGN is professor in toxicology at Department of Cancer Research and Molecular Medicine and AS is a professor in health service research at Department of Public Health and General Practice. All are employed at the Norwegian University of Science and Technology (NTNU), Norway.

## Pre-publication history

The pre-publication history for this paper can be accessed here:

http://www.biomedcentral.com/1472-6882/13/295/prepub

## Supplementary Material

Additional file 1: Table S1: Concomitantly use of herbs and conventional drug-groups. Click here for file
