# Supplementary material for: The co-use of conventional drugs and herbs among patients in Norwegian general practice: a cross-sectional study
Source: BMC Complement Altern Med. 2013 Oct 30;13:295. doi: 10.1186/1472-6882-13-295 (PMC4228482; doi:10.1186/1472-6882-13-295)
Supplement: Additional file 1: Table S1: — Concomitantly use of herbs and conventional drug-groups. [file 1472-6882-13-295-S1.doc]

**Additional file 1: Table S1** Concomitantly use of herbs and conventional drug-groups

|  | Aloe vera | Antho-cyanin | Apple vinegar | Bil-berry | Cinna-mon | Cran-berry | Echin-acea | Garlic | Ginger | Gingko Biloba | Ginseng | GLA/ Evening Primrose oil | Golden root | Grape-fruit | Green tea | Healthy chocolate | Kan Jang | Natto-kinase | Noni juice | Rosehip | Saw Palmetto | Soya extract | Valeriana | Number of combinations |
| --- | --- | --- | --- | --- | --- | --- | --- | --- | --- | --- | --- | --- | --- | --- | --- | --- | --- | --- | --- | --- | --- | --- | --- | --- |
| Against gastrointestinal conditions | 3^A^ |  | 1 | 4 |  | 2 | 2 | 2 | 1 | 2 | 1 |  | 2 | 1 | 4 |  |  |  |  |  | 1 |  |  | 13 |
| Analgesics | 6 | 2 | 3 | 15 | 1 | 9 | 4 | 8 | 2 | 1 | 1 | 1 | 4 | 3 | 11 |  |  | 1 |  |  | 1 | 1 | 1 | 19 |
| Anti-infectives |  |  | 1 | 1 |  | 1 | 1 | 1 |  |  | 1 | 1 | 1 | 1 | 1 |  |  |  |  |  |  |  |  | 10 |
| Anticoagulants | 1 |  |  | 18 |  | 1 | 1 | 9 | 1 |  | 1 |  | 1 | 1 | 7 |  |  |  |  | 1 | 2 | 1 |  | 13 |
| Antidepressants | 2 |  | 1 | 5 |  | 2 | 3 | 3 | 3 |  | 2 | 1 | 2 |  | 5 |  |  |  |  |  |  | 1 | 2 | 13 |
| Antidiabetics | 2 | 1 | 1 | 5 | 1 | 1 | 1 | 4 |  |  | 1 |  | 1 | 2 | 5 |  |  |  | 1 |  |  | 1 |  | 14 |
| Antihistamines | 5 | 1 | 1 | 4 | 1 | 2 | 3 | 3 | 2 | 2 | 1 | 1 | 1 | 1 | 5 |  |  |  |  |  |  | 1 |  | 16 |
| Antihyperlipidemic agents | 5 | 2 | 1 | 2 | 1 | 2 | 5 | 7 | 1 |  | 3 | 1 | 2 |  | 8 |  | 1 |  | 1 | 1 | 2 | 2 | 1 | 19 |
| Antihypertensives and diuretics | 7 | 2 | 3 | 24 | 1 | 5 | 5 | 11 | 3 |  | 2 | 1 | 3 | 2 | 11 |  | 1 |  | 1 | 1 | 1 | 2 | 2 | 20 |
| Anti-menopausal and anticonceptives | 3 | 1 |  | 1 |  | 1 | 2 | 2 | 1 |  |  |  |  |  | 4 |  | 1 | 1 |  | 1 |  |  |  | 11 |
| Antirheumatic | 2 | 1 |  | 5 | 1 | 1 | 1 | 3 | 1 | 1 | 2 | 1 | 2 |  | 5 |  |  |  |  |  |  | 1 |  | 14 |
| Antiseizure. triptanes and central stimulating drugs | 2 | 2 | 1 | 4 |  | 3 | 1 | 3 |  |  | 1 | 1 | 1 | 1 | 3 |  | 1 |  |  |  |  |  |  | 13 |
| Chemotherapeutic drugs |  |  |  | 2 |  |  |  |  |  |  |  |  |  |  | 2 |  |  |  |  |  |  |  |  | 2 |
| Dermal drugs | 2 |  |  | 3 |  | 1 | 1 | 2 |  |  |  |  |  |  | 2 |  |  |  |  |  |  |  | 1 | 7 |
| Drugs against urogenital and prostate disorders | 2 |  |  | 1 |  | 3 |  |  |  |  |  |  |  |  | 1 |  |  |  |  |  | 1 |  |  | 5 |
| Ocular drugs | 2 |  | 1 | 2 |  | 1 | 1 | 1 | 1 | 1 |  |  | 1 | 1 | 1 |  |  |  |  |  | 1 |  |  | 12 |
| Respiratory drugs | 4 |  | 1 | 4 |  | 4 | 1 | 3 | 2 | 2 | 1 |  | 1 | 2 | 3 |  |  |  |  |  |  |  |  | 12 |
| Sedatives and Antipsychotics | 1 |  |  | 8 |  | 4 |  | 6 | 1 |  | 1 |  | 2 | 1 | 6 |  |  |  |  | 1 |  |  |  | 10 |
| Strong analgesics |  |  |  |  |  | 1 |  |  |  |  |  |  |  |  |  |  |  |  |  |  |  |  |  | 1 |
| Thyroids and antithyroids | 4 | 1 | 2 | 6 | 1 | 3 | 4 | 2 | 2 | 2 | 2 | 1 | 2 | 1 | 4 | 1 |  |  |  |  |  | 1 |  | 17 |
| Vasodilators and cardiac glycosides | 1 |  |  | 2 |  |  |  |  |  |  |  |  |  |  |  |  |  |  |  |  |  |  |  | 2 |
| Other drugs | 2 |  | 2 | 4 |  | 1 | 2 | 2 | 1 | 1 | 1 |  | 2 | 2 | 6 |  |  |  |  |  |  |  |  | 12 |
| Number of combinations | 19 | 9 | 13 | 21 | 7 | 20 | 17 | 18 | 14 | 8 | 15 | 9 | 16 | 13 | 20 | 1 | 4 | 2 | 3 | 5 | 7 | 9 | 5 | 225 |

^A^ Highlighted with numbers in bold: Clinical relevant interactions documented in clinical trials, case reports or theoretical interactions extrapolated from clinical data.
